# Premature Birth with Complicated Perinatal Course Delaying Diagnosis of Prader-Willi Syndrome

**DOI:** 10.1155/2011/981941

**Published:** 2011-08-03

**Authors:** G. Ciana, M. C. Fertz, V. Pecile, S. Demarini

**Affiliations:** ^1^Centro di Coordinamento Regionale Malattie Rare, Azienda Ospedaliera Universitaria S. Maria della Misericordia di Udine, Piazzale S. Maria della Misericordia, 33100 Udine, Italy; ^2^S.C. di Neonatologia e Terapia Intensiva Neonatale, IRCCS-Burlo Garofolo, Università degli Studi di Trieste, 34100 Trieste, Italy; ^3^S.C. Laboratorio di Genetica Medica, IRCCS-Burlo Garofolo, Università degli Studi di Trieste, 34100 Trieste, Italy

## Abstract

Prader-Willi syndrome in the newborn is essentially characterized by marked hypotonia, feeding difficulties, hypogonadism, and possible characteristic facial features. However, diagnosis at this age may be particularly difficult, and dysmorphic features may be subtle or absent. Prematurity can furthermore delay clinical features recognition and typical complications due to preterm birth may contribute to divert the diagnosis. We describe a preterm baby with a complicated perinatal course later diagnosed as PWS.

## 1. Introduction

Prader-Willi syndrome (PWS) is a neurobehavioural disorder first described in 1956. Most cases are characterized by neonatal hypotonia, dysmorphic features, short stature, hypogonadism, cognitive impairment, and hyperphagia with subsequent obesity [1]. In newborns, the clinical picture is quite difficult to diagnose, as it differs at this age from that seen later and the dysmorphic features may not always be present [2]. Premature birth may further complicate the recognition of clinical features of PWS and delay the diagnosis, especially if a complicated perinatal course is superimposed. We describe a preterm baby with a complicated perinatal course who was later diagnosed as PWS.

## 2. Case Report

E. L. male, second-born, was delivered at 33 weeks of gestational age by caesarean section because of polyhdramnios and severe intrauterine growth retardation, during pregnancy, referred poor foetal movements. At birth, Apgar scores were 6 and 7 at 1 and 5 min, respectively. Due to persistent hypotonia and hyporeactivity, he was intubated and transferred to the Neonatologic Intensive Care Unit. Birth weight was 1357 gr (<3° centile), length 40 cm (<3° centile), and cranial circumference 29.3 cm (3–10° centile). At admission, some dysmorphic features were evident, as low-set ears, triangular face, high arched palate, bilateral clubfoot, and bilateral cryptorchidism with scrotal hypoplasia. He developed RDS, which was treated with mechanical ventilation and one dose of surfactant. In the first days, a persistent ductus arteriosus was detected and successfully treated with indomethacin. At 1 week of age, he developed a grade 3 intraventricular haemorrhage with progressive evolution to triventricular hydrocephalus; consequently when he was 1 month old an intraventricular reservoir was placed by a neurosurgeon and periodic evacuative injections were necessary. Due to persistence of hypotonia and hyporeactivity, the baby remained intubated and ventilated for 1 month and then received CPAP, as respiratory support for further 20 days. At the same time, he was fed by nasogastric tube due to poor sucking. The global clinical picture was initially explained as a neurological damage in preterm baby with severe intracranial haemorrhage. The persistence of hypotonia coupled with deep areflexia, however, induced us to perform extensive metabolic and instrumental assessments, which turned out negative: hemogasanalysis, lactic acid, urinary organic acids, plasma and urine aminoacidogram, transferrin glycoforms for congenital disorders of glycosylation (CDG), creatine kinase (CK), lactic dehydrogenase (LDH), Brainstem evoked responses, and EEG. Brain MR, beside the triventricular hydrocephalus, showed no other anomalies. A muscular biopsy was not informative. Standard karyotype was normal (46, XY), and FISH for chromosome for Prader-Willi syndrome was negative. 

Neonatal screening for hypothyroidism was doubtful; it was confirmed in our laboratory (TSH 20.56 uU/mL, con fT3 3.72 ed fT4 1.32) and treatment with thyroxine was started. 

When he was 2 months old, the baby was discharged with enteral feeding by nasogastric tube; hydrocephalus showed a stabilization, so either periodic evacuative injections or ventricular-peritoneal shunt were no more necessary. Clubfoot was treated with plaster casts. Subsequently, a progressive start of autonomous sucking was evident. The improvement of hypotonia coupled with cryptorchidism induced us to perform methylation studies at 14 months of life, which turned out positive, revealing the absence of paternally donated imprinting centre indicative of PWS. The study with microsatellites showed the presence of maternal uniparental disomy (UPD). 

## 3. Discussion

Detecting PWS at neonatal age is very important because it allows early intervention and may prevent future problems. However, diagnosis at this age may be particularly difficult, as many of the main features are subtle and evolve in time. Even if approximately 77% of babies with PWS are prematurely delivered [3], the literature concerning prematurity and PWS syndrome is poor, limited to single or few case reports [2, 4, 5] or concerning epidemiological aspects of a PWS general population [6, 7]. So little is known about possible peculiar behaviours and features of the preterm baby with this syndrome. Consensus diagnostic criteria were developed to facilitate the diagnosis of PWS [1] and revised [8]. Concerning the neonatal period, central hypotonia with poor sucking, feeding problems with need for special feeding techniques, hypogonadism and characteristic facial features such as dolichocephaly, almond-shaped eyes, thin upper lip, and narrow bifrontal diameter are the most clinically relevant criteria for detecting PWS. Our patient did not present characteristic facial features of PWS. The lack of typical craniofacial features seems on the other hand to be more frequent in patients with uniparental disomy [9] as our baby, but certainly prematurity with VLBW and developing of hydrocephalus did not facilitate the recognition of eventual dismorphic features. 

He presented hypotonia with poor sucking. This feature is almost universally present at birth [6, 8, 10, 11]; therefore PWS should be considered in the differential diagnosis of all hypotonic newborns [8]. In our patient, however, the confounding factor was the superimposed severe intracranial haemorrhage, otherwise surprising at such a gestational age. It was initially considered responsible for the neurological situation and delayed the investigations. 

The baby needed mechanical ventilation for 1 month and further 20 days of respiratory support. Even in this situation, RDS and then the severe IVH was considered responsible for the necessity of respiratory support, when at a certain time the cause was more probably due to central hypoventilation already described in premature infant with PWS [5]. 

Finally, our first test chosen for diagnose PWS was not correct. This syndrome results from the absence of expression of the paternally active genes in the region 15q11–q13. It results from four mechanisms: a deletion of the critical region q11–q13 of the paternally inherited chromosome 15 (70% of cases), maternal uniparental disomy (UPD), in which two maternal copies of chromosome 15 are inherited, but no paternal copy (20–25% of cases), in <2% from unbalanced translocations involving the PW region and from imprinting defects. Methylation analysis detects all types of molecular defects and nowadays is considered the best screening method to rule out PW. If the methylation pattern is abnormal, then FISH and UPD studies are used to distinguish the type of defect. These tests are the gold standard [12]. Our FISH analysis turned out negative, so diagnosis was further delayed. 

In summary, all hypotonic neonates with feeding difficulties, including premature babies with a disproportionate level of hypotonia for gestational age and need of unexplained prolonged ventilation, have to be tested for PWS, even in absence of typical facial features. Methylation studies should be the first-line exam to perform.
